# Patient Engagement Strategies and Challenges in Patient-Centered Clinical Decision Support: Case Studies From Federally Funded Projects

**DOI:** 10.2196/89911

**Published:** 2026-11-13

**Authors:** Krysta Heaney-Huls, David F Lobach, Sofia Ryan, Kensaku Kawamoto, Courtney D Segal, Priyanka J Desai, CDSiC Implementation, Adoption, and Scaling Workgroup, Deborah Collyar, Prashila Dullabh

**Affiliations:** 1 NORC at the University of Chicago, 1828 L Street NW, 9th Floor, Washington, DC, United States, 1 202-280-9294; 2 Elimu Informatics, El Cerrito, CA, United States; 3 Department of Biomedical Informatics, University of Utah, Salt Lake City, UT, United States

**Keywords:** patient-centered care, clinical decision support systems, patient participation, case study, digital health, mobile health, mHealth

## Abstract

**Background:**

Patient-centered clinical decision support (PC CDS) encompasses a spectrum of decision-making tools that significantly incorporate patient-centered factors related to knowledge, data, delivery, and use. PC CDS supports patient-centered care by helping clinicians and patients make the best decisions based on each individual’s circumstances and preferences. However, effectively engaging patients throughout the PC CDS life cycle remains a challenge.

**Objective:**

This qualitative study examined real-world PC CDS implementation projects. The selected real-world case studies facilitated exploration of patient engagement processes and the identification of challenges and strategies to promote patient involvement, improve PC CDS usability, and facilitate integration into patients’ lifeflows—that is, patients’ daily activities.

**Methods:**

Data were collected through document review and semistructured interviews with researchers from 12 PC CDS projects funded by the Agency for Healthcare Research and Quality. Interview transcripts were analyzed to identify key themes related to patient engagement, challenges, mitigation strategies, and future directions for enhancing patient engagement in PC CDS design, implementation, and adoption.

**Results:**

The study identified 6 patient engagement challenges aligned with 2 different types of patient engagement: engaging patients as partners in co-design (underrepresentation of certain populations among study participants, limited patient participation during group co-design sessions, misalignment of co-design feedback with actionable PC CDS design enhancements, and inadequate staff and resources to synthesize patient feedback) and engaging patients as end users of PC CDS (low adoption of patient-facing technology and limited PC CDS fit into patients’ lifeflows). Our analysis also identified multiple strategies to mitigate these challenges. Mitigation strategies include increasing study representativeness, fostering trust, dedicating multidisciplinary team resources, establishing iterative feedback loops, leveraging user-centered design sessions, promoting co-design, prioritizing ease of use, and using participatory research methods to achieve meaningful patient engagement and enhance tool usability and relevance.

**Conclusions:**

Our findings reinforce the importance of co-design, participatory approaches, and continuous feedback in developing tools that are usable, trusted, and relevant to diverse patient populations. Results also highlight ongoing challenges related to health literacy, digital literacy, representativeness, and long-term engagement. Given growing interest in patient-facing digital health tools, patient engagement will be essential to the successful design, implementation, and adoption of PC CDS.

## Introduction

Patient-centered clinical decision support (PC CDS) encompasses a spectrum of decision-making tools that significantly incorporate patient-centered factors related to knowledge, data, delivery, and use [1]. Within the context of PC CDS, “knowledge” refers to the use of comparative effectiveness research or patient-centered outcomes research (PCOR) findings. “Data” focuses on incorporating patient-generated health data, patient preferences, social determinants of health, and other patient-specific information. “Delivery” refers to directly engaging patients and/or caregivers across different settings. Finally, “use” focuses on facilitating bidirectional information exchange to support patient-centered care, including shared decision-making.

PC CDS researchers, developers, and implementers can encounter a range of challenges throughout the PC CDS life cycle [2]. Common obstacles include issues with data interoperability, integration with existing health information technology (IT) systems, and the maintenance of the privacy and security of patient information [3]. Within the PC CDS life cycle, patients can be engaged both as partners in PC CDS co-design and as end users of PC CDS. These roles are related, as engaging patients in PC CDS design, development, and implementation can improve acceptance but also pose unique challenges in recruiting patients for co-design activities, identifying the best methods for engagement, and translating feedback into intuitive, user-friendly PC CDS that meet the varying needs of patients [4].

Overcoming barriers to patient engagement across key stages of the PC CDS life cycle is necessary to improve adoption and scalability. Previous implementation experiences offer valuable lessons and practical guidance for the PC CDS community regarding the challenges they may encounter and effective mitigation strategies. However, research on patient engagement in PC CDS remains a developing area, with limited aggregation and comparison of challenges across efforts to guide ongoing work.

To address this gap in the literature, we aimed to identify common challenges in engaging patients in the PC CDS life cycle and the strategies used to address them across real-world PC CDS implementation projects.

## Methods

### Overview

This study draws from 12 clinical decision support (CDS) implementation case studies developed from a purposive sample of Agency for Healthcare Research and Quality (AHRQ)–funded projects, including those focused on PC CDS. CDS is a strategic priority area of the Digital Healthcare Research Program, and AHRQ is at the forefront of advancing patient-centered CDS [5]. Specifically in 2016, AHRQ launched a PCOR CDS initiative that initially defined the concept of PC CDS and supported PC CDS development and implementation. Thus, we focused on projects from a portfolio with a concerted focus on patient-centeredness [6].

All members of the analytic team were part of the AHRQ-funded Clinical Decision Support Innovation Collaborative (CDSiC) but were not directly affiliated with AHRQ. To reduce the potential for bias, we implemented triangulation of data sources by using both document review and interviews [7]. In addition, the CDSiC Implementation, Adoption, and Scaling Workgroup, a stakeholder group comprising patients, researchers, developers, and clinicians, informed the case study methods and reviewed the findings. The completed Standards for Reporting Qualitative Research (SRQR) is provided as Checklist 1.

### Project Identification

First, we identified projects from AHRQ’s Digital Healthcare Research Program [8]. We primarily focused on completed projects but also reviewed active projects. We reviewed AHRQ grantee profiles to identify projects aligned with the four patient-centered factors of PC CDS, which are as follows: (1) knowledge, (2) patient data, (3) delivery, and (4) use [1]. From these, we selected a convenience sample of 9 projects that represented a range of care settings, technologies, health conditions, and patient populations (Table 1) [6]. A member of each project team was interviewed to explore PC CDS development experiences, including patient engagement, implementation, adoption, and scaling. After initial analysis, we sought to further explore patient engagement challenges and strategies. To supplement our initial data collection, we identified 3 additional projects from the same AHRQ project portfolio that included patient engagement in the CDS development process. The 3 projects were selected based on discussions among the authors (KK, DFL, KH-H, and PJD) regarding their overall alignment with the 4 patient-centered factors of PC CDS. Project details and information about how each project aligned with the patient-centered factors of PC CDS were abstracted from project documents and summarized in a project profile (Table 1). Profiles were reviewed by AHRQ staff, and study teams were given the opportunity to review project descriptions for accuracy.

**Table 1.** Selected Agency for Healthcare Research and Quality–supported projects.

Project period	Technology type	Condition	Setting	PC CDS^a^ factors^b^
ASTHMAXcel patient-reported outcome (PRO) mobile application to support asthma chronic disease management				
August 1, 2018-May 31, 2022	Mobile app, clinician-facing dashboard	Asthma	Primary care	Knowledge: Asthma guidelines Patient data: Gathers PROs and patient-generated health data Delivery: A patient-facing mobile phone app Use: Facilitates shared decision-making as well as patient self-management through medication reminders and guideline-based asthma education
A Direct-to-Patient Alert for Glycated Hemoglobin Screening Using Prediction Modeling and Mobile Health (mHealth)				
April 1, 2019-March 31, 2022	Text messaging	Type 2 diabetes	Primary care	Knowledge: HbA_1c_ calculator based on American Diabetes Association and US Preventive Services Task Force (USPSTF) screening recommendations [9] Patient data: Predicts HbA_1c_ levels using patient serum HbA_1c_ test data Delivery: Patient-facing text message alerts Use: Recommends patients for screening and orders HbA_1c_ tests if patients agree to diabetes risk screening
Clinical Decision Support for Chronic Pain Management (CDS4CPM)				
September 30, 2019-December 20, 2021	Patient-facing app, clinician-facing app	Chronic pain	Primary care	Knowledge: Centers for Disease Control and Prevention guideline for prescribing opioids for chronic pain [10] Patient data: Consolidates patient-specific data and PROs into a dashboard within the electronic health record (EHR) Delivery: A Substitutable Medical Applications and Reusable Technologies (SMART) on Fast Healthcare Interoperability Resources (FHIR) clinician-facing app and a patient-facing component Use: Supports shared decision-making around chronic pain management
Enabling Shared Decision Making to Reduce Harm from Drug Interactions: An End-to-End Demonstration				
September 30, 2019-September 29, 2021	SMART on FHIR app	Gastrointestinal bleeding due to DDIs	Clinics with anticoagulation services	Knowledge: Validated anticoagulant drug-drug interaction (DDI) clinical decision support (CDS) artifacts [11] Patient data: Integrates patient risk profile with EHR workflow Delivery: A SMART on FHIR clinician-facing app Use: Facilitates shared decision-making around the risks of DDIs
Implementing USPSTF Recommendations for Breast Cancer Screening and Prevention by Integrating Clinical Decision Support Tools with the Electronic Health Record^c^				
September 30, 2020-August 31, 2025^d^	SMART on FHIR app	Breast cancer	Preventive care	Knowledge: USPSTF guidelines for breast cancer screening and prevention [12] Patient data: Incorporates patient-generated risk factors and EHR data into a screening tool Delivery: A SMART on FHIR patient-facing and clinician-facing app Use: Facilitates shared decision-making, providing personalized risk assessments and educational resources
Patient Outcomes Reporting for Timely Assessments of Life with Depression: PORTAL-Depression				
September 30, 2019-September 29, 2021	Patient portal	Major depressive disorder	Primary care	Knowledge: USPSTF screening guidelines for depression in adults [13]; computerized adaptive test for mental health [14] Patient data: Gathers patient-reported symptoms of depression Delivery: A patient portal questionnaire with results stored in EHR for the clinician Use: Informs clinical monitoring of at-risk patients screened for depression
Scalable Decision Support and Shared Decision Making for Lung Cancer Screening				
July 1, 2019-present^d^	Clinician-facing EHR integrated app	Lung cancer screening	Primary care and pulmonary clinics	Knowledge: USPSTF guidelines for lung cancer screening [15] Patient data: Incorporates patient-specific lung cancer risk factor information from the EHR Delivery: A SMART on FHIR clinician-facing Use: Promotes shared decision-making by displaying individualized benefits and risks of lung cancer screening
Scaling Interoperable Clinical Decision Support for Patient-Centered Chronic Pain Care				
September 13, 2021-August 31, 2025^d^	EHR dashboard, patient-facing app	Chronic pain	Primary care	Knowledge: Evidence-based patient-centered outcomes research (PCOR) findings related to chronic pain [16] Patient data: Incorporates patient-reported data on pain and physical function Delivery: A SMART on FHIR patient-facing and clinician-facing app Use: Promotes shared decision-making between clinicians and patients by aiding in treatment plan selection
Shareable, Interoperable Clinical Decision Support for Older Adults: Advancing Fall Assessment and Prevention Patient-Centered Outcomes Research Findings into Diverse Primary Care Practices (ASPIRE)				
August 1, 2020-December 31, 2022	Clinician-facing EHR app	Fall prevention	Primary care	Knowledge: Evidence-based decision rules for fall risk assessment in primary care Patient data: Uses patient fall-risk screening data Delivery: A SMART on FHIR clinician-facing app Use: Shares care plan collaboration tool to address fall risk factors
The Impact of an Adaptive Patient-Centered Web Application on Medication Optimization in HFrEF Patients^c^				
September 30, 2022-September 29, 2025^d^	SMART on FHIR app	HFrEF	Primary care, specialty care	Knowledge: Validated HFrEF medication optimization algorithm [17] Patient data: Incorporates patient-specific health data from the EHR Delivery: A SMART on FHIR patient-facing app Use: Facilitates shared decision-making
Translating Hypertension Guidelines into Practice: Development of Interoperable Clinical Decision Support^e^				
September 30, 2019-December 31, 2021	Patient portal app, EHR-based dashboard	Hypertension	Primary care	Knowledge: 71 recommendations from 8 hypertension guidelines [18] Patient data: Incorporates patients’ blood pressure (BP) history, patients’ BP goals, medications, prior adverse drug events, and recommendations based on patients’ data and clinical guidelines Delivery: A SMART on FHIR patient-facing app Use: Engages patients in controlling high BP
The Wise App Trial for Improving Health Outcomes in PLWH^c^				
September 30, 2020-August 31, 2025^d^	Mobile app	HIV/AIDS	Patient home	Knowledge: Evidence for chronic disease self-management and medication adherence in people living with HIV Patient data: Incorporates patient-specific health information and medication adherence data from a linked smart pillbox and user input into the app Delivery: A patient-facing mobile phone app Use: Enhances patient self-management and improves antiretroviral therapy adherence

a PC CDS: patient-centered clinical decision support.

b “Knowledge” refers to the use of comparative effectiveness research or PCOR findings. “Patient data” focuses on the incorporation of patient-generated health data, patient preferences, social determinants of health, and other patient-specific information. “Delivery” refers to directly engaging patients and/or caregivers across different settings. “Use” focuses on facilitating bidirectional information exchange in support of patient-centered care, including shared decision-making.

c Second-round case studies that included patient engagement as part of PC CDS development.

d Indicates an active project.

e HBP: high blood pressure.

### Document Review

For all projects, we reviewed publicly available project documents, including profiles on the AHRQ website, final reports, publications, and public webinars. In total, we reviewed 44 documents, including 9 final reports, 26 published papers, 5 AHRQ project files, and materials from 5 webinars. From these sources, we abstracted information about project characteristics (study setting type of PC CDS, and specifics about the technology intervention), types of patient engagement activities, information about how patient feedback was used, and information about challenges (eg, challenges associated with recruitment, facilitation, health literacy/digital health literacy, or patient adoption of PC CDS), strategies (ie, activities projects undertook to address documented challenges), and future recommendations.

Using a matrix literature review approach, 3 researchers (KH-H, SR, and CDS) qualitatively identified information gaps for each project to inform the development of customized semistructured interview guides (see the following section). In addition, we identified themes across projects in three predefined categories: (1) addressing challenges arising from patient context and experience, (2) overcoming barriers to incorporating patient input across all stages of the PC CDS life cycle, and (3) enhancing engagement with PC CDS to improve its adoption.

### Qualitative Interviews

Qualitative interviews were used to supplement and contextualize findings from the document review. We created a semistructured interview guide, and for each interview, the guide was customized based on preliminary data gathered during the document review to focus discussions on topics that were not addressed in publicly available documents (eg, how patient input was used in the project and challenges in facilitation approaches) and to gather additional information on challenges and strategies identified through the document review. Guides included questions that reflected the full PC CDS life cycle (ie, design, implementation, adoption, and scaling).

In the first phase of case studies, we conducted key informant interviews with project leaders (n=9) using a semistructured interview guide that probed project experiences related to patient engagement, implementation, adoption, and scaling. For the second phase (n=3), the semistructured interview guide was modified to expand upon patient engagement topics identified in the first phase, including challenges related to addressing health and digital literacy, incorporating patient feedback, and aligning PC CDS with patients’ daily activities, or “lifeflows” [19]. Each hour-long interview was conducted via web conference and recorded with participant consent. We created interview transcripts to accurately capture participants’ comments for analysis.

We used a qualitative framework matrix approach to analyze interview transcripts [20]. For each project interview (“cases”), 3 researchers (KH-H, SR, CDS) reviewed the transcripts and extracted data into predefined columns (“codes”) for challenges, strategies, and future directions related to patient engagement in PC CDS design, implementation, or adoption (ie, 9 columns in total).

### Synthesis Across Information Sources

To converge the data sources, the document review matrices and interview matrices were combined, enabling data across information sources to be presented side by side for synthesis [21]. Using the combined matrix, we further synthesized findings qualitatively within and across projects for the three predefined categories established during document review: (1) addressing challenges arising from patient context and experience; (2) overcoming barriers to incorporating patient input across all stages of the PC CDS life cycle; and (3) enhancing engagement of PC CDS to improve adoption. Using qualitative content analysis, we synthesized these 3 predefined categories into 2 broader thematic areas of challenges: engaging patients as partners in co-design and engaging patients as end users of PC CDS. Under each thematic area, we inductively identified subthemes for each challenge and associated strategies [22]. Disagreements regarding thematic synthesis within these areas were adjudicated by PD, PJD, KK, and DFL, and all findings were reviewed by the CDSiC Implementation, Adoption, and Scaling Workgroup.

### Ethical Considerations

This study was reviewed by the National Opinion Research Center at the University of Chicago Institutional Review Board and did not meet the definition of research with human participants set forth in the Federal Regulations at 45 CFR 46.102 [23].

## Results

### Overview

Our analysis identified 6 patient engagement challenges that project teams encountered or mitigated during PC CDS design, implementation, and adoption. These challenges include designing user-friendly interfaces, addressing health literacy, and meeting patient needs and preferences. We categorized these challenges to correspond with two facets of patient engagement: (1) engaging patients as partners in PC CDS co-design and (2) engaging patients as end users. While these challenges represent long-standing barriers to meaningful patient engagement in research and care [1], the potential impact on uptake, use, and patient-centered care can be amplified in the context of PC CDS. We also documented over 2 dozen strategies implemented or suggested to address these challenges (Tables 2 and 3).

**Table 2.** Co-design challenges and mitigation strategies.

Challenges to engaging patients as partners in co-design	Strategies to engage patients as partners in co-design
Underrepresentation of certain populations among study participants, including patients with varying levels of health and digital literacy	Deliberately recruit patients from medically underserved populations Offer financial incentives to compensate patients for their time Offer in-person and web conferencing–facilitated options to engage patients with differing levels of digital literacy
Limited patient participation during group co-design sessions	Use methods that enable one-on-one interactions with patients Foster patient confidence to contribute during the co-design process by ensuring that feedback is collected in a nonjudgmental and supportive environment
Misalignment of focus group feedback with actionable PC CDS^a^ design enhancements	Prepare simulated clinical scenarios that exemplify the purpose and intended use of the PC CDS Involve informatics and usability experts in co-design activities to provide information on the feasibility of patient recommendations and to offer alternatives
Inadequate staff and resources available to synthesize patient feedback to inform PC CDS enhancements	Assemble multidisciplinary teams to support PC CDS design and development Dedicate sufficient time, staffing, and financial resources to conduct and leverage user-centered design feedback sessions Establish feedback loops that allow the project team to inform patients about how their feedback shaped the PC CDS

a PC CDS: patient-centered clinical decision support.

**Table 3.** End-user engagement challenges and mitigation strategies.

Challenges to engaging patients as end users of PC CDS^a^	Strategies to engage patients as end users
Low adoption of patient-facing technology (eg, patient portals)	Identify opportunities to use multiple delivery modalities for the PC CDS (eg, app or web browser) Engage patients by making the adoption process as simple as possible, such as by simplifying technical instructions using plain language, providing user guides, using incentives, and ensuring the tools are integrated seamlessly with other health information technology systems
Limited PC CDS fit into patients’ lifeflows	Ask patients to identify when and how to integrate patient-facing clinical decision support into their lifeflows Integrate co-design into PC CDS design and development Apply human-centered design principles in PC CDS design and development Use participatory research methods in PC CDS design and development Conduct systematic usability assessments during design and development Measure health literacy and make necessary changes based on the results of these assessments to ensure patients can understand and use PC CDS Monitor usage and follow up with patients with low usage to determine if PC CDS use is impacted by usability issues that can be addressed Catalyze clinicians to promote use among their clinical teams and patient populations Use reminder emails to reinforce the intervention and communication of recommendations encouraging patients to engage in shared decision-making at their upcoming visit Plan for other differences within the intended patient population (eg, sex, socioeconomic status, non-English language preferences)

a PC CDS: patient-centered clinical decision support.

### Challenges in Engaging Patients as Partners in Co-Design

We identified 4 challenges related to engaging patients in co-design for PC CDS development (Table 2).

#### Underrepresentation of Certain Populations Among Study Participants, Including Patients With Varying Levels of Health and Digital Literacy

Achieving patient representativeness in PC CDS studies is important to ensure that study findings are generalizable to the intended patient populations. Failure to do so can lead to incomplete feedback from potential end users that would otherwise inform implementation of the CDS Five Rights [24]. However, recruiting representative study populations adds complexity to study designs.

One project team cited limitations in the generalizability of their patient perspective analysis, which aimed to gather input on adapting CDS recommendations and patient-facing decision support visualizations. Study participants were younger and less representative of underserved populations than people in the United States with the condition [25]. A second project team noted that the patient population in their health system had a smaller proportion of Black/African American individuals compared with the US population. Project teams also reported difficulty recruiting patients with lower levels of digital literacy for patient co-design sessions facilitated via web conferencing, noting that those who volunteered were more comfortable with computers and other digital technologies. Additionally, the proliferation of web conferencing options since the COVID-19 pandemic may bias participation toward individuals with higher levels of digital literacy.

Project teams emphasized the importance of recruitment methods that engage patients from a broader range of demographic groups to participate in PC CDS co-design. In particular, the PC CDS community should develop approaches to recruit and engage individuals with lower health and digital literacy, individuals representing underserved populations, and people representing a range of patient characteristics. This is especially relevant for PC CDS designed to address health concerns that differentially affect populations with higher incidence and risk of disease, such as HIV [26]. In these cases, researchers would benefit from gathering input and preferences from a range of patients about how guideline recommendations do or do not fit into their health care decision-making.

One project team advocated providing financial compensation to patient participants. They noted that this approach can improve the recruitment of a more representative range of patient participants, particularly when barriers to participation are related to a patient’s socioeconomic status.

To facilitate recruitment of patients with lower levels of digital literacy, one project team suggested offering both in-person and web conferencing options for patient participation.

#### Limited Patient Participation During Group Co-Design Feedback Sessions

Patient-centered co-design helps ensure that decision support tools meet the needs of end users. While well-established methods exist to engage clinicians in co-design, methods to engage patients in co-design are emergent [27]. Project teams used a range of methods for patient-centered co-design, including focus groups, individual interviews, surveys, simulated patient use cases, and participatory design sessions.

Focus group discussions can help identify differences and similarities across patient perspectives, including preferences and end-user needs, which CDS developers can use to refine decision support functional requirements [28]. However, one project team expressed concerns that some participants may have felt overwhelmed by more vocal members of the groups and may have found it difficult to share their opinions. Another team observed that the patients recruited to participate in usability testing, which included simulated clinical scenarios and a semistructured interview, had less familiarity with the expectations and principles of patient participation in research, which may have limited their degree of comfort in sharing their feedback.

To mitigate the risk of focus group findings disproportionately representing the voices of more vocal participants, PC CDS implementation teams can use methods that enable one-on-one interactions with patients, such as individual interviews or think-aloud sessions instead of focus groups, roundtable sessions, or other group discussion–based co-design activities.

To foster a supportive and safe environment that helps patients feel comfortable, co-design facilitators should solicit feedback in a nonjudgmental way. PC CDS implementation teams should reassure patients that their feedback is valued and be prepared to answer questions about how patient feedback will be used. Another project used competition methods between patients in discussion groups to make the process of providing feedback more fun and engaging for patients. A project team member described this process:

*...during these design sessions we had people break out into groups and the groups kind of became competitive against each other of like who could come up with the best design...really nice to see that people were that engaged, that they actually were competing on who’s going to come up with the best design at the end of an hour and a half.*

#### Misalignment of Focus Group Feedback With Actionable PC CDS Design Enhancements

While the goal of co-design is to gather end-user feedback on functional requirements, 2 project teams shared that patients may not focus their feedback on the PC CDS itself or may have trouble understanding the process and context in which the PC CDS will be delivered.

To maximize meaningful feedback, one project team found success by preparing simulated clinical scenarios (ie, a description of the patient health care encounter that replicates a real-life situation in which the PC CDS would be used) for patients to review in advance to help ground the discussion on developing the CDS. Another team invited IT experts to join co-design sessions with patients to provide input on the feasibility of implementing patients’ suggestions. Finally, another project had human factors experts lead co-design sessions with clinicians—a model that could be replicated for patient engagement activities.

#### Inadequate Staff and Resources Available to Synthesize Patient Feedback to Inform PC CDS Enhancements

Incorporating patient feedback into PC CDS is critical to ensure that the tools meet patients’ needs and preferences while providing the right information to the right patient at the right time. Failure to do so could affect adoption, use, and effectiveness.

Analyzing data from patient engagement activities requires qualitative researchers to interpret and translate patient feedback for PC CDS developers to use in tool design. Extensive resources in terms of expertise and time may be needed to synthesize and incorporate patient feedback. For example, one project team indicated that the analysis of patient feedback required researchers with expertise in both qualitative methods and CDS development across various settings. Additional time and resources were needed to compile findings and synthesize lessons learned so that CDS developers could modify the tool. A different project team stated that, in retrospect, it would have allocated more time and resources to test patient interview guides to better focus patient feedback on the PC CDS. Another stressed the value of engaging patients in co-design as early as possible. However, project budgets and timelines may constrain the funding and time available to incorporate the suggested changes.

Therefore, PC CDS project leaders should assemble multidisciplinary teams that work with a range of patient groups to develop PC CDS. These teams should also include user-centered design experts and informaticians with expertise in PC CDS integration to ensure that the input aligns with the project scope and can be implemented.

To address challenges related to involving patients early in the co-design process and to establishing feedback loops that communicate how patient feedback was used in PC CDS co-design and development, researchers should consider including these activities in grant applications and project protocols to ensure that sufficient time, staffing, and monetary resources are allocated to conduct and leverage iterative user-centered design feedback sessions.

### Challenges in Engaging Patients as End Users

We identified 2 challenges related to engaging patients as PC CDS end users (Table 3).

#### Low Adoption of Patient-Facing Technology

One adoption challenge identified was the limited reach of digital tools such as patient portals, particularly when the portal is the only means of engaging patients in PC CDS. One project team found that many patients either did not respond to or did not engage with the portal, despite multiple outreach attempts.

To overcome this challenge, the project team adapted the PC CDS by offering a tablet-based version in the waiting room. However, this method still relied on the patient being present and willing to engage with the tool. The team acknowledged that although offering the PC CDS in various formats improved access, it was insufficient to reach the “nonresponders” who may not actively engage with technology.

To address low adoption more broadly, project teams encouraged developers to ensure seamless integration of PC CDS with existing health IT systems. Another team emphasized ease of use, the development of intuitive interfaces, and simple user instructions. Plain language can also promote accessibility for patients with varying levels of digital literacy. Additional support, such as step-by-step guides, can also be offered to those who need guidance, including caregivers or patient navigators. A project team member stated, “we built...a very simple user interface...[and] design elements that would lend themselves to [a more intuitive experience]...to get [patients] to that final step of seeing the results.” Finally, PC CDS implementers could consider using incentives to increase adoption.

#### Limited PC CDS Fit Into Patients’ Lifeflows

We define use as the incorporation of a PC CDS into both clinical workflows and patients’ lifeflows—namely, the intersection of activities patients engage in outside a health care encounter and the PC CDS [29]. Limited PC CDS use reduces the potential impact of interventions on desired behavior change and health outcomes.

Two project teams encountered uptake challenges that were attributed, in part, to PC CDS functionality that was not well integrated into the patients’ lifeflow. Patient representatives and project teams reported reduced use because the timing of alerts and reminders and their frequency could not be customized by patients. In one instance, clinical notifications were generated by clinician activity, but the timing did not account for the impact on the patient. In another instance, patients reported they received clinical recommendations at inconvenient times during the day, limiting their ability to act on them.

To address low uptake and the potential impact on effectiveness due to misalignment between features and functionality, and patients’ needs, PC CDS developers should account for how PC CDS integrates into patients’ lifeflows. A range of co-design and participatory research methods that involve stakeholder participation and collaboration across the research cycle can improve the validity of PC CDS design and development and ensure that PC CDS fits into patients’ lifeflows.

Several projects used participatory research methods to identify the optimal time to engage patients via PC CDS, particularly when engagement occurs outside the clinical encounter and allows patients to modify it based on their preferences (eg, frequency of use and complexity of patients’ health needs). For example, 4 projects used participatory research methods with patients, including participatory design sessions [30], think-aloud sessions, and cognitive walk-throughs [31], in the early stages to inform PC CDS design.

One project reported leveraging a consultative patient advisory group to participate in formative co-design processes, validating its PC CDS design ideas and providing input on prototype designs. Another project used an iterative, human-centered design implementation framework to guide evaluation of its PC CDS development. Key elements of the framework include convening multistakeholder groups across the spectrum of design and development activities, including issue identification; defining the vision and goals of the intervention and PC CDS; usability testing and user-informed design enhancements; and implementation evaluation, which includes continuous feedback and refinement to improve the user experience. Additionally, researchers can systematically assess usability. For example, researchers should consider identifying a prespecified threshold for acceptable usability scores, using performance-based usability metrics, and assessing this cut point alongside other qualitative techniques such as think-aloud sessions and cognitive walk-throughs [32].

*I think people actually like using our tools once they're in the trial. I think for us, it feels like it’s worth all these processes...I think people generally appreciate that the goal of this is to try to design and develop tools that are to help people within their population. And I think they're generally interested in engaging in the process. However, a different research member reflected, There’s probably not enough community-based participatory research in CDS research*.

[Project team member]

Another component of PC CDS integration with patients’ lifeflows is ensuring the PC CDS provides guidance and health information that patients can understand and use to support decision-making and self-care actions. Thus, addressing “digital health literacy”—defined as “the ability to seek, find, understand, and appraise health information from electronic sources and apply the knowledge gained to address or solve a health problem” [33]—can improve use. PC CDS developers and implementation teams can also use health literacy assessments and health communication best practices to ensure patients can understand and use PC CDS-provided information and recommendations. Several project teams proactively incorporated strategies to address health literacy into their study designs, such as using health literacy assessments to measure differences in literacy and education levels among study participants. Other projects emphasized the importance of using plain language for PC CDS user instructions and messages to support shared decision-making. Project teams also advocated that PC CDS developers identify opportunities to develop content in other languages to reduce participation barriers for patients with non-English language preferences.

Two projects highlighted the importance of monitoring usage and following up with patients who showed low engagement. One project surveyed clinical teams and patients to gain insight into usability barriers. Low usage was often linked to issues with the PC CDS design or functionality, which could be addressed through iterative improvements. Active support from clinical teams also played a crucial role in improving engagement. Having support from the clinical teams was useful for identifying these adoption barriers and for promoting the tool within the clinic and among patients. Reminder emails were another effective strategy for boosting engagement with the PC CDS. These emails reminded patients of recommendations and encouraged shared decision-making discussions during upcoming clinic visits.

Finally, PC CDS developers should try to account for other differences among the intended patient population, such as socioeconomic status and sex [34], which could impact adoption and use.

## Discussion

Patient engagement is a critical aspect of PC CDS. However, implementing processes that meet patients where they are and reflect patient preferences regarding participation can be challenging. Despite these challenges, researchers and PC CDS developers recognize the importance of patient engagement and are increasingly incorporating patient co-design into their projects. For each of the challenges described above, the projects in our case study highlight promising patient engagement strategies that others can use.

### Principal Results

Our case studies identified several patient engagement challenges and strategies to address them that others in the field can implement as part of their work. Patient engagement through co-design and iterative feedback is critical in creating PC CDS that is usable, effective, and trusted by patients. Ensuring that PC CDS is accessible to a wide range of users, particularly those with varying levels of digital and health literacy, is essential for successful adoption. Strategies such as feedback loops, which allow patients to see how their input has influenced the design, further enhance trust and engagement. Meaningful patient engagement should incorporate processes that empower patients, support their understanding of PC CDS, and enable them to contribute to the design and development of PC CDS activities [35].

However, challenges such as health literacy remain. Project teams highlighted the need to better engage patients with lower health literacy when designing PC CDS, especially tools that are patient portal–based. This can begin during the recruitment process by tailoring recruitment materials for people of differing health literacy levels, such as using plain language and visuals [36]. During PC CDS development, IT or CDS experts can participate in patient co-design sessions to provide information on the feasibility of patient recommendations. Additionally, given that patients with lower health literacy may feel uncomfortable in group discussion settings, researchers should consider using a wider range of data collection modalities (eg, individual interviews) to ensure patients feel empowered to give feedback.

Project teams also pointed out that patient engagement can be difficult to sustain over time, particularly in vulnerable populations [37]. Using more sustained methods, such as convening patient advisory groups throughout implementation to gather patient feedback, may be a useful next step to identify approaches for long-term engagement with PC CDS.

### Comparison With Prior Work

Previous work emphasizes that meaningful patient engagement for PC CDS should (1) support cooperative relationships among patients and clinicians, health care organizations, and PC CDS developers; (2) provide opportunities for patients to participate in iterative and sustained feedback activities in a way that is appropriate to the individual; and (3) underscore empathy for end users’ expertise and life experiences to inform health care decisions that are important to the patient [38,39]. Consistent with the previous literature, our findings highlighted the importance of co-design with patients to create these cooperative relationships. In particular, co-design can help improve the patient-centeredness of PC CDS by ensuring patients’ needs are accurately reflected [27], which ultimately facilitates shared decision-making processes and reinforces trust in PC CDS [1,40].

The projects also underscored the importance of ensuring that PC CDS meets patients’ needs to promote adoption and use. While this work did not explore specific methods to achieve this, it is important to note that previous suggestions in the literature include identifying and incorporating into PC CDS features that align with patients’ digital health literacy levels to improve accessibility. For example, including user guides or “info buttons” within PC CDS can provide additional guidance and direction on PC CDS use [31]. Additionally, during PC CDS implementation, health systems could consider providing training or educational tools for patient populations who need more assistance in using PC CDS.

Finally, while this work focused on patient engagement in the design and implementation of PC CDS, previous work emphasizes the importance of patient engagement upstream in the development of clinical guidelines that serve as the knowledge source for PC CDS [41-43]. A multidisciplinary approach to guideline development that includes patients is needed to encourage standardization, reduce redundancies, and ensure that guidelines are usable by both clinicians and patients [43]. The patient engagement strategies highlighted in our case studies may be applicable to this process. For example, recruiting patients from underserved populations and facilitating the engagement of patients with varying levels of health and digital literacy during the guideline development process will ensure that the guidelines are relevant and acceptable to a range of patient groups, thus facilitating their acceptance and use in patient care. Additionally, dedicating sufficient time and resources for user-centered feedback sessions with patients during clinical guideline development can be useful in addressing social, behavioral, and environmental factors [27].

### Limitations

This study has limitations that should be considered when interpreting the findings. First, we selected projects from AHRQ’s portfolio of PC CDS projects, given its focus on patient-centeredness and patient engagement. However, we recognize that a broader pool of real-world PC CDS implementation projects may have generated strategies not captured in this work. Second, we aimed to select projects that involved a range of patient populations and conditions to help capture a variety of patient engagement challenges and approaches. However, most of the projects took place in primary care settings, which could limit generalizability to other care settings. Finally, some of the studies assessed in our evaluation were in the early stages of implementation, so our findings may not reflect the entirety of patient engagement activities and lessons learned.

### Conclusions

A central takeaway from the case studies is the importance of involving patients early *and* throughout the design process to ensure the tools meet their needs and preferences. Our findings point to the need for more patient-driven approaches in future PC CDS development and more research to explore how to sustain long-term engagement with PC CDS throughout their development and implementation. Notably, several project teams in our study developed patient-facing mobile apps to help patients manage their care, identify whether preventive screening is needed, and facilitate shared decision-making with their clinician. These tools encourage patient-centeredness as they allow patients to easily access information related to their care on their phones, outside of a regular clinical encounter. Given the growing interest in the patient-facing health technology ecosystem, patient-centered co-design approaches will be key to the successful integration of patient-facing digital health tools into patients’ lifeflows, supporting shared decision-making, and enhancing the quality of care.

## Supplementary material

10.2196/89911 Checklist 1 SRQR checklist.

## Abbreviations

AHRQ: Agency for Healthcare Research and Quality
CDS: clinical decision support
CDSiC: Clinical Support Innovation Collaborative
IT: information technology
PC CDS: patient-centered clinical decision support
PCOR: patient-centered outcomes research
SRQR: Standards for Reporting Qualitative Research
